# Illuminating the Role of Vpr in HIV Infection of Myeloid Cells

**DOI:** 10.3389/fimmu.2019.01606

**Published:** 2019-07-23

**Authors:** Sarah Beth Nodder, Suryaram Gummuluru

**Affiliations:** Department of Microbiology, Boston University School of Medicine, Boston, MA, United States

**Keywords:** HIV, Vpr, myeloid, DDR, DCAF, VprBP, ubiquitin ligase

## Abstract

Vpr is a 14 kDa accessory protein conserved amongst extant primate lentiviruses that is required for virus replication *in vivo*. Although many functions have been attributed to Vpr, its primary role, and the function under selective pressure *in vivo*, remains elusive. The minimal importance of Vpr in infection of activated CD4+ T cells *in vitro* suggests that its major importance lies in overcoming restriction to virus replication in non-cycling myeloid cell populations, such as macrophages and dendritic cells. HIV-1 replication is attenuated in the absence of Vpr in myeloid cells such as monocyte-derived dendritic cells (MDDCs) and macrophages, and is correlated with the ability of Vpr to overcome a post-integration transcriptional defect in these cells. Intriguingly, recent identification of the human hub silencing (HUSH) complex as a target for DCAF^CRL4^-mediated degradation by numerous ancestral SIV Vpr alleles, and the Vpr paralog Vpx, signifies the potential function of HIV-1 Vpr to alter yet-to-be identified chromatin remodeling complexes and prevent host-mediated transcriptional repression of both invading viral genomes and pro-inflammatory responses. Myeloid cells constitute an important bridge between innate and adaptive immune responses to invading pathogens. Here, we seek to illustrate the numerous means by which Vpr manipulates the myeloid cellular environment and facilitates virus replication, myeloid cell-dependent HIV transmission, and systemic virus dissemination.

## Introduction

Sexual transmission is the predominant means by which HIV is acquired (“Global AIDS Update” 2016). While the exact cell type targeted by HIV in the genital mucosa remains a matter of debate (1–3), various subsets of dendritic cells (DCs) and macrophages are present at high concentrations within the genital mucosa, and therefore may be early targets of HIV (4–6). Infection of DCs and macrophages is particularly important as they are uniquely poised to transmit HIV with high efficiency to CD4+ T cells during antigen presentation within the secondary lymphoid organs (5, 7, 8). As professional antigen presenting cells, DCs and macrophages have a unique cellular architecture to initiate and sustain robust interactions with CD4+ T cells. Virological synapse formation between DCs /macrophages and CD4+ T cells ensures directed delivery of HIV to its most permissive host: activated CD4+ T cells (9). In order for mucosal transmission and establishment of productive infection, HIV not only has to navigate tissue barriers (3), but also a number of cell-intrinsic immune defenses, or restriction factors, such as APOBEC3G (a cytidine deaminase that dramatically increases genome mutations), tetherin (which prevents HIV viral budding and enforces a positive type I IFN loop upon suppression of viral budding), and SAMHD1 (a dNTPase that limits dNTP levels within the cytoplasm to hinder reverse transcription), that prevent primate lentiviral infection of DCs, macrophages and resting T cells (10–13). However, primate lentiviruses have evolved to counteract these restriction factors by encoding accessory proteins that selectively inhibit their anti-viral function. Although postulated to act in this capacity, the function of Vpr has yet to be fully understood.

Vpr, or viral protein R, is a 14 kDa protein, encoded by all extant primate lentiviruses. Vpr is actively packaged into virions through its interaction with the p^6^ region of Gag (14, 15), and as such has roles in both pre- and post-integration steps of the viral life cycle. Although originally described as an accessory protein, and thus dispensable for virus replication *in vitro*, it has since been shown to play an important role in the infection of macrophages and dendritic cells (16–19). Importantly, Vpr function is necessary for viral pathogenesis *in vivo*. In 1993, six rhesus macaques were infected with pathogenic SIV_mac_239 isolate either containing or lacking Vpr (20). Within 16 weeks, wild type Vpr sequences were isolated from three of the five animals infected with Vpr null virus. Moreover, the remaining two animals infected with Vpr null virus displayed delayed pathogenesis, and reversion to Vpr-expressing virus by 66 weeks (20, 21). These studies validated retrospective work that found reversions of an internal Vpr stop codon to an open reading frame in an accidentally infected laboratory worker and experimentally infected chimpanzees (22, 23). Together, these studies were seminal in igniting research into the role of Vpr in the pathogenesis of HIV. Vpr is best known to induce an Ataxia-Telangiectasia and Rad3-related (ATR) dependent-DNA damage response, or DDR (24). Vpr-mediated DDR activation results in a G_2_ to M cell cycle arrest in cycling cells, which is perplexing as the cell populations whose infections are seemingly most reliant on the presence of Vpr are terminally differentiated and thus not susceptible to cell cycle arrest. The ability to induce a G_2_ to M cell cycle arrest is thought to be advantageous for viral transcription as the HIV LTR has been shown to be most active during this phase of the cell cycle (23, 25). What's more, the Vpr residues that confer G_2_ to M cell cycle arrest capabilities are under positive selection *in vivo* and are thus the most well-studied in the field (26). It remains to be determined if induction of DDR by Vpr, an evolutionarily conserved function amongst all primate lentiviral Vpr proteins (27–29), is necessary for establishment of virus replication in metabolically quiescent immune target cells, such as monocytes, macrophages and DCs.

### Vpr/Vpx and the Importance of Co-opting the Ubiquitin-Ligase DCAF^CRL4^ in Myeloid Cells

Vpx, or Viral protein x, arose following the duplication of Vpr post-diversion of the primate lentiviral lineages that gave rise to HIV-1 and HIV-2 (30, 31). Vpx has a well-characterized role in degrading the retroviral restriction factor SAMHD1 (10, 11). In terminally differentiated or non-cycling cells, SAMHD1 reduces the concentration of dNTPs within the cytoplasm, thereby drastically limiting reverse transcription (32–35). Vpx bridges SAMHD1 to the E3-ubiquitin ligase CUL4A-DDB1 DCAF (DCAF^CRL4^) leading to its polyubiquitination and proteasomal degradation (10, 11, 36). There has been much interest in identifying the restriction factor(s) targeted by Vpr as it similarly co-opts the DCAF^CRL4^ complex to ubiquitinate target host proteins. Unlike Vpx however, infection of myeloid cells by either HIV-1 or HIV-2 still occurs in the absence of Vpr, albeit with significantly different outcomes (19, 24, 37–41). It is likely that the replication advantage conferred by Vpr lies in its ability to induce a DDR, though the mechanisms by which Vpr-induced DDR facilitates enhanced virus replication and spread *in vivo* are still to be determined. The multitude of DDR proteins associated with the Vpr-DCAF^CRL4^ complex (24, 37–39, 41), suggests that Vpr by co-opting a host protein complex involved in multiple cellular pathways, has managed to maximize its impact at the interface of virus and host to promote HIV spread.

### Vpr Residues Involved in DCAF^CRL4^ Engagement

An NMR structure of HIV-1 Vpr provides insight into how it interacts with multiple proteins. Both N and C-termini are unstructured (nucleotides 1–16, and 77–96, respectively) and flank three α-helices from nucleotides 17–33, 38–50, and 56–77 (42). The HIV-2 Vpr, as well as the closely related Vpr alleles from SIV_smm_ and SIV_mac_, are predicted to be structurally homologous to that of HIV-1. Whilst the unstructured C- and N-terminal domains facilitate interactions with host targets, the DCAF^CRL4^- binding domain is isolated to the third α-helix (42–45). The HIV-1 Vpr mutants Q65R and H71R for example, and corresponding residues in HIV-2/SIV_mac_ Vpr alleles fall within this region and fully abrogate Vpr-DCAF^CRL4^ interactions. These mutations prevent Vpr-mediated transcriptional enhancement in MDDCs (19), decrease degradation of multiple DNA damage response proteins (46–50), and prevent G_2_/M cell cycle arrest in CD4+ T cells (51). Furthermore, ablation of Vpr-DCAF^CRL4^ interaction, as occurs with a VprQ65R mutation (albeit not with the VprQ77R mutation), has been associated with long-term non-progression *in vivo* (52). Investigations into the DCAF^CRL4^-mediated enhancement of infection in myeloid cells use these select mutations to infer mechanisms of action and are thus worthy of mention.

## DCAF^CRL4^-Dependent Roles of Vpr in Myeloid Cells

### DNA Damage Response Proteins

#### Human Uracil DNA Glycosylase

Monocyte-derived macrophages (MDMs) have a high ratio of dUTP/TTP in their cytoplasm that can lead to the mis-incorporation of uracil in the reverse transcribed genome. The ratio of dUTP/TTP in macrophages was found to be as high as 60 (53, 54). Human uracil DNA glycosylase (hUNG) excises mis-incorporated UTP and recruits additional repair enzymes to the site of genome mutation. Thus, HIV-1 Vpr-mediated DCAF^CRL4^-dependent ubiquitination and proteasomal degradation of hUNG was hypothesized to restrict virus replication through either degradation of uracilated viral DNA prior to integration or via transcriptional interference of the uracilated provirus (53, 55, 56). However, due to the intrinsically low levels of hUNG in MDMs (56), the utility of uracil-dependent restriction of HIV-1 in MDMs is limited. Furthermore, infection of MDDCs with HIV-1 expressing hUNG-binding deficient VprW54R mutant does not result in transcriptional attenuation nor deficiency in viral spread (19). Thus, the significance of hUNG degradation by the Vpr-DCAF^CRL4^ complex (39, 57) remains unclear.

#### SLX4-SLX/MUS81-EME1

The importance of Vpr in the Holliday junction repair pathway has been of great interest as it promised to provide insight into the role of Vpr at the viral integration step. Original reports suggested that HIV-1 Vpr-DCAF^CRL4^-mediated ubiquitination of MUS81 which, in the presence of phosphorylated EME1 and kinase-active PLK1, prematurely activates the quaternary endonuclease complex SLX4com (47). This activation was shown to precede G_2_/M cell cycle arrest and result in the formation of FANCD2 foci as a result of activation of the Fanconi anemia pathway (47). Notably, virion-associated Vpr-mediated activation of SLX4com was shown to prevent type I IFN production which is of great importance due to the myriad of interferon stimulated genes (ISGs) that modulate myeloid cell function and determine the dissemination efficiency of virus through the host (47). However, subsequent studies have only shown a Vpr-DCAF^CRL4^ dependent degradation of the SLX4com subunits MUS81-EME1 (50, 58–60) and have not addressed whether the active SLX4com suppressed innate immune detection of HIV-1 in myeloid cells. Additionally, interaction of SLX4com with Vpr is not conserved amongst all primate lentiviral Vpr alleles (59). Together, published findings so far, suggest that the Vpr-mediated activation of SLX4com does not have a conserved role in suppressing innate immune detection of primate lentiviruses in myeloid cells.

#### Helicase-Like Transcription Factor (HLTF)

Helicase like transcription factor, or HLTF, is a target of Vpr-mediated DCAF^CRL4^ degradation (46, 48). Like UNG2 and SLX4-SLX1/MUS81-EME1, HLTF is involved in DNA damage repair. Specifically, HLTF is critical to the remodeling and repair of stalled replication forks (61). Although HLTF is degraded in macrophages in a Vpr- DCAF^CRL4^ dependent manner, it is unclear whether HLTF antagonizes viral replication in myeloid cells.

#### Exonuclease 1

Exonuclease 1, or Exo1, is a Rad2/XPG 5′ to 3′ exonuclease involved in numerous DNA repair processes that ensures genome stability throughout the cell cycle (62). Exo1 has recently been identified as a substrate for Vpr-DCAF^CRL4^ polyubiquitination and proteasomal degradation in CD4+ T cells (49). The authors speculate that Exo1 antagonism prevents deleterious processing of reverse transcription- and viral integration-intermediates, and thereby attribute Exo1 restriction to virion-associated Vpr rather than its *de novo* synthesized partner (49). As of yet, Exo1 has not been shown to play a role in promoting HIV-1 infection of macrophages or dendritic cells.

While these published studies highlight the numerous interactions of Vpr with diverse DDR proteins, contribution of these interactions to viral pathogenesis have remained unclear. Although understudied in the case of HIV-1 infection, there is a robust literature tying innate immune signaling and DDR (63, 64). It should be noted that manipulation of the DDR is not unique to HIV. Rather, it is a shared pathogenic strategy used extensively at the interface of hosts with both bacteria and viruses that can promote pathogen replication and pathogenesis (65). Kaposi sarcoma herpesvirus, for example, encodes a protein (Latency-Associated Nuclear Antigen or LANA) that sequesters Rad50, Mre11, and NBS1, all members of the DDR signaling activator MRN complex to prevent cytoplasmic sensing of viral DNA and innate immune activation (66). Another example of virus subversion of DDR pathway includes murine γ-herpesvirus which specifically encodes orf36 whose role is to induce an ATM-dependent DDR and H2AX phosphorylation (67). In the absence of orf36 or ATM activation, virus replication is attenuated, pointing toward a role for the DDR in facilitating virus replication (67). Overall, it is evident that Vpr uses DCAF^CRL4^ to induce a DDR, with potentially divergent outcomes in different cell populations. What remains unclear is how activation of the DDR and interaction of Vpr with DNA repair proteins allows viral evasion of immune detection in myeloid cells. Since the kinetics of reverse transcription of HIV-1 in myeloid cells is relatively slow, it is tempting to speculate that manipulation of diverse DDR pathways is a conserved strategy by primate lentiviral Vpr alleles to overcome premature host repair of viral reverse transcription intermediates (63), though definitive evidence for this hypothesis has been lacking. Rather, it is likely that activation of DDR promotes multiple discrete stages of the virus life cycle. For example, Vpr can induce DDR through both the ATM and ATR pathways (24, 68). Unresolved ATM activity can lead to activation of NF-κB (69) and increased production of inflammatory cytokines, such as IL-6, both of which can result in enhanced viral gene expression and macrophage-dependent HIV-1 transmission to CD4+ T cells (70).

### Vpr Functions in Transcriptional De-repression

#### Transcriptional Enhancement

Previous work by our group has shown a post-integration defect in monocyte-derived dendritic cells (MDDCs), infected with Vpr deficient HIV-1 (19). Infections in the absence of virion associated Vpr were characterized by low proviral transcription despite similar levels of integration, and reduced infection of CD4+ T cells in co-cultures (19). This defect is dependent on Vpr binding to DCAF^CRL4^ as it is fully abrogated upon infection with Vpr mutants (Q65R or H71R) lacking DCAF^CRL4^ interactions. It should be noted that numerous viruses besides HIV-1, most notably Hepatitis B virus, can also manipulate the E3 ubiquitin ligase DCAF^CRL4^ to enhance transcription (71). While, the mechanism of HIV-1 Vpr-mediated transcriptional enhancement remains unclear, previous research has shown Vpr-mediated degradation of HDACs (38) and members of the NuRD chromatin remodeling complex (72) which may globally enhance transcription. Furthermore, DCAF^CRL4^ also has a well-known role in the degradation of a transcriptional repressor, ATF3, which is necessary to correct UV-damage (73). This explanation is not satisfactory given the cell type dependency of the transcriptional enhancement. Whether a MDDC-specific repressor/activator is degraded or sequestered remains unknown and warrants further investigation.

#### TET2

Members of the TET DNA dioxygenase family have recently been shown to be degraded in a Vpr-DCAF^CRL4^ dependent manner (70). In myeloid cells TET2 is naturally monoubiquitinated. TET2 N-terminal monoubiquitylation allows for efficient binding to chromatin and subsequent recruitment of chromatin remodeling machinery and transcription factors. However, in the presence of Vpr, TET2 is rapidly polyubiquitinated at a site independent of its natural monoubiquitylation site and undergoes DCAF^CRL4^- dependent proteasomal degradation (70). This is relevant in myeloid cells as TET2 is an upstream suppressor of IL-6 expression. TET2 recruits HDAC1 and HDAC2 to the IL-6 promotor thereby repressing IL-6 transcription. Importantly, in monocyte-derived macrophages and the monocytic cell line THP-1, the lack of Vpr-mediated degradation of TET2 was associated with reduced viral particle release and slower spread of HIV-1 infection. Upon TET2 knockout, the differences in infection between Vpr-competent and Vpr-deficient viruses was lost. Since IL-6 has long been recognized as a transcriptional enhancer of HIV in monocytes (70, 74–76), these findings are further suggestive of a direct link between Vpr, TET2 degradation, and persistent IL-6 production, which might result in enhanced efficiency of viral spread from myeloid cells to CD4+ T cells.

#### Epigenetic Regulation of Provirus

Until recently, it was not known whether there were host-intrinsic mechanisms to restrict retroviral replication following integration. However, recent investigations have identified a novel method of cell-intrinsic restriction: that of deposition of transcriptionally suppressive methylation marks at proviral LTRs. Following reverse transcription and integration, the LTR of proviruses within heterochromatin are methylated through the sequential recruitment of HP1 and the methyltransferase Suv39H1 (77) Tri-methylated H3K9 recruits the HUSH (HUman Silencing Hub) complex of which there are three components; TASOR, MPP8, and periphilin (78). Although the HUSH complex does not harbor methyltransferase activity itself, HUSH recruits the methylase SETDB1 which induces further H3K9me3 methylation of the provirus. Notably, shRNA-mediated knockdown of each HUSH complex protein rescues endogenous and exogenous retroviral gene expression, thereby signifying the importance of its quaternary assembly for transcriptional repression (78). Interestingly, TASOR is targeted by the SIVmac/HIV-2 lineage Vpx for DCAF^CRL4^-mediated polyubiquitination and proteasomal degradation, thereby increasing the transcriptional activity of proviruses that would otherwise be suppressed (78–80). While HUSH complex can repress transcription from integrated HIV-1 LTR (78, 80, 81), surprisingly, HIV-1 Vpr does not target the HUSH complex proteins for degradation (79). However, multiple Vpr alleles from ancestral primate lentiviruses to HIV-1, including alleles derived from SIV_AGM_, SIV_MUS2_, and SIV_SAB_, have been shown to prevent HUSH-mediated silencing (79, 80). These studies mark the beginning of investigations into Vpx/Vpr antagonism of antiviral host proteins at the proviral DNA level. Although HUSH-mediated transcriptional silencing is not a myeloid specific anti-viral mechanism, the HUSH complex and its associated facilitators are active in myeloid lineages. Recent studies in the literature provide evidence for epigenetic control of proinflammatory cytokine responses in macrophages (82, 83). For instance, the histone methyltransferases, SETDB1 and Smyd2, potently suppress TLR4- mediated induction of IL-6 and TNFα production, and mice with macrophage-specific SetDB1 deficiency are hyper-responsive to endotoxin challenge (82). Whilst antagonism of HUSH complex has not been attributed to HIV-1 Vpr, transcriptional silencing of the HIV-1 LTR in MDDCs in the absence of Vpr (19) suggests the existence of additional mechanisms of myeloid cell-intrinsic transcriptional repression that are targeted by HIV-1 Vpr.

#### Dicer and miRNA Processing

Modulation of the RNA interference (RNAi) and microRNA (miRNA) pathways is an integral means by which pathogens usurp host functions to their advantage (84, 85). MicroRNAs in particular have long been known to play a role in HIV replication in multiple cell populations. For instance, miR-29a, has been implicated in the suppression of HIV mRNA levels through its binding to the 3′-UTR of HIV RNA and subsequent attachment to P body proteins and RISC complexes (86). Dicer is required for processing pre-miRNA substrates to reveal a double-stranded miRNA complex, which then binds to the RISC complex and represses target mRNA expression, either via translation inhibition or via mRNA degradation (87). Recent studies have also demonstrated the ability of miRNAs to negatively regulate proinflammatory responses in macrophages by restricting chromatin remodeling and enforcing transcriptional silencing of promoters of select inflammatory genes (88). Interestingly, Dicer has been identified in complex with Vpr-DCAF^CRL4^ prior to its degradation and depletion of Dicer within infected MDMs has been shown to increase viral replication via unknown mechanisms (89). We posit that Vpr-Dicer dependent modulation of select miRNA expression might contribute to the de-repression of inflammatory responses. It should be noted that Vpr-mediated Dicer depletion has also been shown in CD4+ T cells and as such, is not a myeloid-specific antagonist of innate restriction (89). The role of Dicer degradation has yet to be fully understood, particularly as research into the function of miRNA and RNAi in HIV pathogenesis is increasing (90).

## DCAF^CRL4^-Independent Roles of Vpr in Myeloid Cells

### Envelope Trafficking

Myeloid cells often populate mucosal tissues and as such are poised to disseminate HIV from the periphery to sites harboring abundant activated CD4+ T cells. Macrophages and dendritic cells are capable of transferring virus via cis or trans infection. Both methods facilitate the concentration of virions to the infectious synapse, and in doing so greatly increase the probability of CD4+ T cell infection (9, 91, 92). Two studies have investigated the role of Vpr in the concentration and delivery of virus at the virological synapse. Both of these studies show that in the absence of Vpr, Env-positive virions are trafficked to the lysosome for degradation, thereby reducing the efficiency of macrophage to CD4+ T cell virus spread at low multiplicity of infection (93, 94). In contrast, our investigations into whether Vpr facilitates evasion of this Env-dependent reduction in virus release in other cells types, such as MDDCs, have not yielded similar results (19), suggesting that the effect of Vpr on Env expression might be restricted to specific cell types and not universally observed.

### Type I IFN and Pro-Inflammatory Responses

There is mounting evidence to suggest that Vpr modulates the immune response of myeloid cells to favor viral replication and dissemination throughout the host. Early studies suggested a possible defect in the activation of MDMs and MDDCs upon treatment with recombinant Vpr (95). This defect was characterized by low CD33 surface expression, poor CD80/86 upregulation, and impaired antigen presentation to activated CD4+ T cells (95). In contrast to studies utilizing exogenous addition of recombinant Vpr, there has been a preponderance of research investigating the role of Vpr in the context of a viral infection. For instance, *de novo* expression of Vpr in productively infected MDDCs induced pro-inflammatory cytokine (TNF-α, IL-6 and IL-8) production (70, 96, 97). Previous work by our group has shown enhanced proviral transcription in MDDCs in the presence of Vpr (19). Other studies have similarly shown a role for Vpr in proviral transcription. Liu et al. showed that Vpr alters the availability of the NF- κB p50-p65 heterodimer and AP1 (98), both of which are necessary for the initiation of HIV transcription from the 5′ LTR (99, 100) and expression of pro-inflammatory cytokines. In this study, Vpr was shown to facilitate the polyubiquitination and subsequent phosphorylation (activation) of TAK1, an upstream regulator of NF-κB and AP1 (98). Interruption of TAK1 phosphorylation, and thus inhibiting its activation, significantly reduced proviral transcription (98).

Both our study (19) and the work showing Vpr-mediated modification of TAK1 (98) are important in light of the recent identification of a novel viral detection pathway: one in which host sensing of *de novo* expressed intron-containing HIV-1 RNA (HIV icRNA) in MDMs and MDDCs results in ISG expression and proinflammatory chemokine and cytokine production, including IP-10 and IL-15 (101). IP-10 is an inflammatory chemokine and is a ligand for the receptor CXCR3 (102), while IL-15 is a γ-chain cytokine critical for the proliferation and homeostasis of T cells (103). CXCR3 is expressed on activated CD4+ T cells, and thus secretion of IP-10 from productively infected myeloid cells may result in additional recruitment of virus-susceptible cells to sites of viral replication in the peripheral tissues. Furthermore, IL-15 exposure can result in SAMHD1 phosphorylation and inactivation of its dNTPase activity, thus alleviating restrictions to viral replication in quiescent CD4+ T cells (104). Interestingly, a TAK1 inhibitor reduced production of IP-10 from HIV-1 infected macrophages (101). Thus, in this model, Vpr-dependent enhanced proviral transcription potentially increases the pool of viral icRNAs subject to cell-intrinsic innate immune sensing, resulting in the establishment of a pro-inflammatory state and enhanced virus dissemination.

It should be noted that an increase in viral transcription in myeloid cells is a double-edged sword for HIV in that there is also induction of ISG expression and establishment of a putative anti-viral state. While the nucleic acid sensing mechanism responsible for detection of HIV icRNA is yet to be determined, Vpr can block phosphorylation and nuclear translocation of interferon regulatory factor 3 (IRF3) via inducing ubiquitination and proteosomal degradation of IRF3, though this has only been shown in CD4+ T cell lines (105). Additionally, Vpr has been shown to bind to TBK1 in myeloid cells and prevent its phosphorylation, thereby preventing induction of type I IFN production (106). Thus, we posit that Vpr might function to promote NF-κB-dependent pro-inflammatory responses while contributing to the suppression of induction of anti-viral host defenses. Furthermore, induction of ISGs such as CD169 in MDMs and MDDCs upon host sensing of HIV icRNA in myeloid cells (101) might further tip the balance toward enhanced virus dissemination as opposed to virus restriction. For instance, induced CD169 expression on HIV-infected macrophages and dendritic cells can facilitate cell-to-cell transmission of CD4+ T cells across infectious synapses (101, 107, 108). Together, these studies point to the role of Vpr as a protein that carefully navigates multiple viral sensing systems to induce recruitment of additional cellular targets of virus, whilst evading antiviral immunity.

## Conclusion

**Figure 1:**
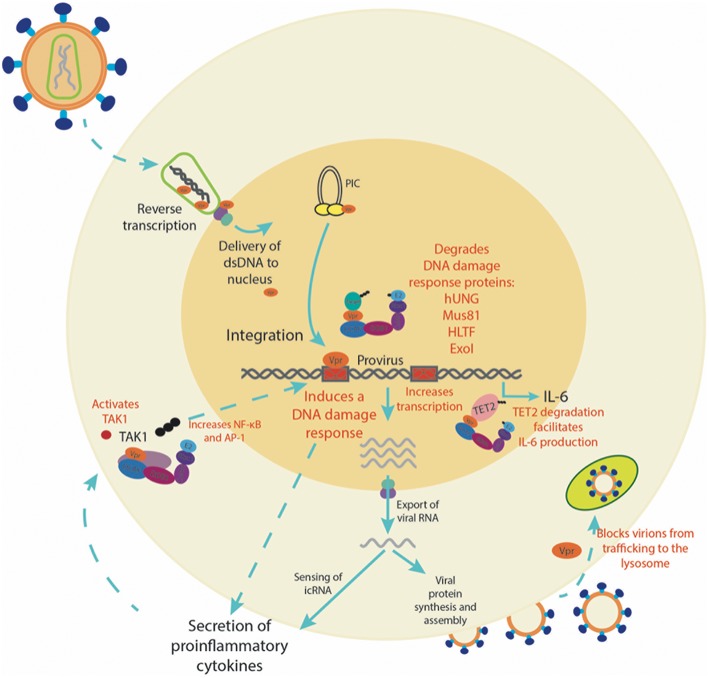
The role of Vpr in the infection of myeloid cells. A summary of the multiple functions Vpr plays in myeloid cells, from enhancing transcription and inducing a DDR to the secretion of pro-inflammatory cytokines. In red are processes in which Vpr has been directly investigated.

It is clear that Vpr plays an important role in the infection of myeloid cells (see Figure 1). A number of tissue-resident macrophages, such as microglia, kupfer cells, alveolar, intestinal, testicular and vaginal macrophages harbor proviral DNA (109–113), and tissue-resident macrophages are estimated to compromise up to 4% of infected cells *in vivo* (114), and importantly, can remain persistently infected with HIV-1 even in the presence of cART (109–112, 115). It is possible that the Vpr-mediated DDR activates a pro-inflammatory state that promotes the establishment of a tissue-resident myeloid cell reservoir, whereby virus spreads efficiently due to persistent virion production and enhanced cell-to-cell contacts between HIV-infected myeloid cells and CD4+ T cells. In this way, the infection of myeloid cells is the bridge between the relatively hostile sites of virus acquisition (most notably the peripheral mucosal tissues) and the key target of HIV; CD4+ T cells. It seems likely that the true value of Vpr *in vivo* is its versatility, allowing for evasion of viral restriction both prior to and post integration in myeloid cells. Future studies will need to address the relative importance of each of the known Vpr functions *in vivo*.

## Author Contributions

All authors listed have made a substantial, direct and intellectual contribution to the work, and approved it for publication.

### Conflict of Interest Statement

The authors declare that the research was conducted in the absence of any commercial or financial relationships that could be construed as a potential conflict of interest.
